# A Cross-National Validation of the Shortened Version of the Adolescent Stress Questionnaire (ASQ-S) Among Adolescents From Switzerland, Germany, and Greece

**DOI:** 10.3389/fpsyg.2021.619493

**Published:** 2021-04-09

**Authors:** Beyhan Ertanir, Christian Rietz, Ulrike Graf, Wassilis Kassis

**Affiliations:** ^1^Institute Research and Development, University of Applied Sciences and Arts Northwestern Switzerland, Windisch, Switzerland; ^2^Department of Educational Science, Heidelberg University of Education, Heidelberg, Germany

**Keywords:** adolescence, stress assessment, cross-national, measurement invariance, validity, reliability

## Abstract

The experience of stress is receiving increasing attention in the context of adolescent mental health, which is why a valid and reliable stress assessment instrument is of great importance. For this purpose, an English-language adolescent stress questionnaire (ASQ) was developed, which assesses the subjective stress experience of adolescents in different areas of life (e.g., at home, at school, and during leisure time). However, the latest long version of the questionnaire with 56 items (ASQ-2) was found to be too extensive, so a more economical short version ASQ-S with 27 items was developed. The aim of this study was to validate a German and a Greek version of the ASQ-S. In order to investigate the psychometric properties of the German and Greek ASQ-S confirmatory factor analysis, analyses of variance and correlations were applied to sample data from Switzerland, Germany, and Greece (*N* = 1,071 seventh-grade students; *M*_*age*_ = 12.53; *SD* = 0.76). The results yielded only poor to moderate internal reliability across all three countries and the suggested 9-dimensional factor structure could not be confirmed. Instead, a modified 6-factor structure was tested which showed acceptable model fits while demonstrating form invariance across the three countries. Furthermore, the ASQ-S scales correlated positively with depressive symptoms and anxiety and negatively with self-esteem and life satisfaction, all of which supported adequate concurrent validity. The results revealed that the utility of the ASQ-S appears to be limited when translated to other languages and should be used with caution when administered in international contexts.

## Introduction

Adolescence is characterized by a significant increase in strain and stress in different life spheres (Hilt et al., 2010; Skinner and Zimmer-Gembeck, 2016). In fact, the transition from childhood to adolescence often goes hand in hand with rapid changes in home, school, and social context (Santrock, 2004). Simultaneously, it leads to substantial changes at the level of individual development. Young people are challenged not only by sexual and physical changes, but also by their targeted preparation for work life and the pressure to gain access to future employment opportunities (Seiffge-Krenke, 2019). These profound changes in several contexts lead to an increasing burden of potential stress factors such as conflicts with parents, pressure to adapt to peers, general school and performance related stress, and fear of the future (e.g., Phillips et al., 2019). High stress levels are considered a risk factor for various behavioral and emotional disorders, psychosomatic problems, and other illnesses in adolescence (Seiffge-Krenke, 2000; Compas et al., 2014; Calvete et al., 2015; Zimmer-Gembeck et al., 2016). Concern is raised in the Western world in particular because findings indicate that diverse psychological disorders such as antisocial behavior, substance abuse, and depression, frequently occur during adolescence (Sawyer et al., 2000).

A national survey in Switzerland showed that about 50% of Swiss adolescents reported being highly stressed in daily life, while only 14% claimed to be never or rarely stressed (Jacobs Foundation, 2015). Similarly, an international study also revealed a decline in adolescent mental well-being in many European countries between the years 2014 and 2018. The results also demonstrated that the experience of suffering from mental health issues differs across countries (Inchley et al., 2020). For example, differences were found between Germany and Greece in terms of adolescents’ reports of feeling low, nervous or irritable. Among 15-year-olds, the prevalence of feeling low more than once a week was twice as high in Greece as in Germany (Inchley et al., 2020). Likewise, Greek adolescents aged between 11 and 15 reported higher rates of irritability and nervousness compared to their counterparts in Germany and Switzerland.

The unavoidability of stress in everyday life, the high prevalence rates, and the variations across countries all point to the importance of examining the stress experience in different countries. There is especially a need for appropriate measures that meet the standards of psychometric quality and capture common and contemporary stressors. The current study focuses on typical, everyday stressors that frequently occur in adolescence.

An often-used and internationally well-established measure is the adolescent stress questionnaire (ASQ) (Byrne and Mazanov, 2002), which exists in different long- and short-form versions. The latest long (56-item) version of the questionnaire captures the subjective stress experience of adolescents at home, at school, in their free time, in romantic peer relationships, in situations of peer pressure, in interaction with teachers, and in relation to finances, as well as regarding their own visions of the future and their experience of responsibility for their own lives (Byrne et al., 2007). The long version of the instrument was validated on a sample of 1,240 adolescents from Germany, Hungary, Spain, Greece, and Denmark (De Vriendt et al., 2011). The reliability parameters were found to be low (test–retest reliability) and overly moderate (internal consistency). For the whole sample, it was reported that only 50% of the stress component scales have Cronbach α-values of ≥0.8, ranging from α = 0.57 to α = 0.88 and only two out of 10 scales have Test–retest ICCs ≥ 0.7, ranging from 0.45 to 0.85. In addition, it was visible that the factor structure could not be proven consistently (De Vriendt et al., 2011).

In the meantime, the ASQ has been translated into many languages (e.g., Dutch, Greek, Hungarian, Norwegian, Spanish, Swedish, and Turkish) and has already been used worldwide. The results regarding the psychometric properties are mixed. While studies from Australia (Byrne et al., 2007), Greece (Darviri et al., 2014), New Zealand (Sotardi and Watson, 2019), Spain (Lima et al., 2017), and Turkey (Tanış, 2019) were able to show that the factor structure fits well for the long version, there are other studies that raise remarkable concerns regarding the structural validity and internal consistency (Moksnes and Espnes, 2011), test–retest reliability (De Vriendt et al., 2011), and problematic factor loadings of the ASQ (McKay et al., 2016). However, the majority of the previous work describes it as a valid and reliable instrument of stress measurement, and one which also seems to work in different cultures. The fact that short versions of the ASQ exist also helps to make it very attractive. Short versions are exceptionally well-suited for research purposes as well as for use in the clinical context due to their short implementation time. For this reason, two short versions, differing in their number of items and subscales, were developed at the same time. While Anniko et al. (2018) suggested a shortened version of the ASQ (ASQ-S) with 27 selected items capturing nine scales, Andretta et al. (2018) proposed an eight-scale short version of the ASQ based on 24 items. Both short versions indicate adequate psychometric criteria. Nevertheless, a validation study from United Kingdom (McKay et al., 2019) points to problems of the 27-item version (Anniko et al., 2018). More specifically, the authors McKay et al. (2019) broach the issue of limited internal consistency. However, the problem of lack of consistency of some scales also existed in the prior long form versions. In addition, it is also visible that problems in terms of concurrent validity exist. Most of the correlation coefficients of the ASQ-S scores and criterion variables are below *r* = 0.50 and thereby showing considerably lower values when compared with the full ASQ scores. In summary, the picture regarding the psychometric properties of ASQ is still unclear and illustrates the need for further validation studies in different languages and countries.

In the current study, we want to investigate the cross-national validity of the German and Greece translations of the ASQ-S proposed by Anniko et al. (2018) and to replicate the suggested 9-factor structure in three European countries. More specifically, the aim of the present study is to examine the model fits, internal consistencies, and the concurrent validity of the ASQ-S in different countries (Anniko et al., 2018). To our knowledge, there are no existing studies in which the ASQ-S has been used in Germany, Switzerland, and Greece. Therefore, it is important to further validate the instrument in order to determine whether the suggested model is sensitive for language translations and whether it works in different countries. For the purpose of assessing the factor structure, we used confirmatory factor analyses (CFA) and cross-validated the factor structure across the countries. An examination of measurement invariance across countries was used to investigate whether the instrument measures the intended latent construct equivalently across contexts and groups (Chen, 2007). In previous studies the ASQ and ASQ-S have been validated with measures of self-esteem, depression, anxiety, and life satisfaction. The concurrent and construct validity was supported by different studies for the long and short versions as well (e.g., Lima et al., 2017; McKay et al., 2019). In the current study, we also use measures of self-esteem, depression, anxiety, and life satisfaction to prove the concurrent validity of the German and Greek translations.

## Materials and Methods

### Sample and Procedures

The data used in our study derives from a sample of 1,071 seventh-grade students in 119 classrooms (74 schools) who took part in an international longitudinal study conducted in Switzerland (German-speaking parts), Germany, and Greece in autumn 2019. The overarching goal of the study was to examine school resilience patterns of middle school aged students.

The German-speaking adolescents’ sample was collected from randomly contacted schools in Northwestern Switzerland (Cantons Aargau, Basel-City, and Solothurn) and the German state Baden-Württemberg. The Greek sample was recruited from different schools of the cities Athens, Chania, and Larissa. The head masters and teachers of the schools were informed about the goals of the research and were asked to participate in the study. Schools assented to participate provided classes consisting of students aged 11–20 years (*M* = 12.53; *SD* = 0.76). Each school dealt with the process of informed consent and parental permission. Students were given a standardized introduction to the survey which informed them of its purpose and gave them instructions on how to complete the online questionnaire using tablets. All students present on the data collection day and who indicating their willingness to participate completed the anonymous questionnaires in class during a regular school day, within an average of 45 min.

The Swiss data set consisted of 349 (boys: *n* = 183 (54%); *M*_*age*_ = 12.6, *SD* = 0.69) 12- to 15-year-old students, whereas the German participants were 322 (boys: *n* = 179 (43%); *M*_*age*_ = 12.74, *SD* = 0.77) 12- to 15-year-olds. In Greece a total of 400 (boys: *n* = 168 (56%); *M*_*age*_ = 12.18, *SD* = 0.70) eligible adolescents, ages 11–20 years took part in the study.

### Measures

#### Stress Experience

The ASQ-S (Anniko et al., 2018) was translated into German and Greek using a four-eyes principle technique, a content translation process, with the help of native speakers in each language. The instrument consists of nine scales with different numbers of items ranging from 2 to 4 items per scale. The scales refer to adolescents’ perceived level of stress comprising different stressors in the domains of home life, school performance, school attendance, romantic relationships, peer pressure, teacher interaction, future uncertainty, school/leisure conflict, and financial pressure. Respondents were asked to indicate their stress experience on a scale ranging from 1 = *not stressful at all* to 5 = *highly stressful*. The reliability of the scales was estimated with Cronbach’s α and is reported in Table 1.

**TABLE 1:** Table of means, standard deviations, and test score reliability of ASQ-S (9-factor solution).

	CH		GER		GR				Reliability				Reliability		
	*n* = 349		*n* = 322		*n* = 400				CH	GER	GR		CH	GER	GR
	Mean	SD	Mean	SD	Mean	SD	*F*	Cohen’s f	Cronbach’s α/				Cronbach’s α/		
									Spearman-Brown				Spearman-Brown		
									Coefficient				Coefficient		
F1: Home Life	2.60	1.19	2.59	1.26	2.79	1.16	2.78	0.08	0.80	0.84	0.77	F1: Home Life	–^2^	–^2^	–^2^
F2: School Performance	3.15	1.02	3.21	1.05	3.12	1.04	0.59	0.03	0.68	0.66	0.65	F2: School Life	0.83	0.83	0.83
F3: School Attendance	2.29	1.06	2.59	1.15	2.07	1.21	16.74***	0.19	0.69^1^	0.65^1^	0.75^1^	F3: Romantic Relationships	–^2^	–^2^	–^2^
F4: Romantic Relationships	1.70	0.87	1.86	1.02	2.74	1.34	52.45***	0.42	0.69^1^	0.65^1^	0.70^1^	F4: Peer Pressure	–^2^	–^2^	–^2^
F5: Peer Pressure	2.54	1.05	2.59	1.07	2.87	1.16	6.45**	0.14	0.80	0.74	0.81	F5: Future Uncertainty	–^2^	–^2^	–^2^
F6: Teacher Interaction	2.70	1.01	2.70	0.99	2.57	1.01	1.83	0.06	0.70	0.58	0.50	F6: Financial Pressure	–^2^	–^2^	–^2^
F7: Future Uncertainty	3.35	1.13	3.21	1.06	3.06	1.11	4.95**	0.12	0.57^1^	0.60^1^	0.71^1^				
F8: School Leisure	2.69	0.99	2.78	1.06	2.87	1.08	2.52	0.07	0.69	0.63	0.62				
F9: Financial Pressure	2.48	1.20	2.42	1.27	2.54	1.18	0.52	0.05	0.71^1^	0.81^1^	0.78^1^				
Total ASQ-S	2.66	0.75	2.70	0.79	2.81	0.78	3.53*	0.08	0.94	0.94	0.93				

CH = Switzerland, GER = Germany, GR = Greece; *SD* = Standard Deviation, *F* = *F*-Test (ANOVA statistic), *post hoc* test for country differences (Tukey, significant at the 5% error level). ***p < 0.001, **p < 0.01, and *p < 0.05. ^1^Spearman-Brown coefficients were calculated for two-item subscales. ^2^Same as the statistics for the 9-factor solution.

#### Life Satisfaction

The adolescents’ life satisfaction was assessed using The Satisfaction with Life Scale (SWLS) (Diener et al., 1985). The usefulness of the existing German version of the scales had previously been confirmed in representative German-speaking samples (Diener et al., 1985). The Greek version was also obtained through a four-eye principle translation process. Adolescents were asked to rate their life satisfaction on a 1 to 7 point-Likert scale ranging from *strongly disagree* to *totally agree* via five items. The Cronbach’s α in this study was α = 0.83 (CH: α = 0.83, GER: α = 0.79, GR: α = 0.87).

#### Self-Esteem

*The Rosenberg Self-Esteem Scale* (Rosenberg, 1965) was used to assess the students’ self-esteem. Using 10 items, participants were asked to indicate on a four-point Likert scale the degree to which statements applied to them (from 1 = *strongly disagree* to 4 = *strongly agree*). Internal consistency for the present sample was α = 0.77 (CH: α = 0.82, GER: α = 0.78, GR: α = 0.72).

#### Depression and Anxiety

The German and the Greek translations of the HSCL-25 (Derogatis et al., 1974) were used to assess symptoms of depression and anxiety. Students were asked to indicate how much they had been bothered by 10 symptoms of anxiety and 14 symptoms of depression during the past week. The item “Losing sexual interest” was excluded from the item list. Response categories ranged from 1 (=*not at all*) to 4 (=*very much*). Internal consistencies for the subscales varied between α = 0.85 (anxiety; CH: α = 0.86, GER: α = 0.83, GR: α = 0.87) and α = 0.91 (depression; CH: α = 0.93, GER: α = 0.93, GR: α = 0.89).

### Statistical Analyses

To determine the cross-national differences in the mean levels of stress experience of adolescents, mean scores of the all items for each subscale were computed. Country differences in the mean levels were tested using an ANOVA. Subsequently, *post hoc* tests (Tukey) were conducted to determine which group scored higher than the other one(s) on a particular stress dimension. Internal consistency was assessed by calculating Cronbach’s α or Spearman-Brown coefficients for scores on each factor and the total score on the ASQ-S. Descriptive analyses and the variance analyses were performed using the SPSS 24.0 statistical software package.

Confirmatory factor analyses were conducted with *Mplus* (Muthén and Muthén, 1998-2017) using maximum likelihood estimation in order to assess the factor structures. To account for missing data (<5% for all items) the Full Information Maximum Likelihood Estimation-Option (FIML) was applied. We decided to use the maximum likelihood estimation as other methods generally exhibit greater biases than *Missing at Random* approaches (Graham, 2009; Enders, 2013). In order to address any non-normality in the data, Maximum Likelihood Robust (MLR) estimator was used. Extreme cases were only detected for item 15 (Getting along with your teachers).

The chi-square statistic can be used for assessing the goodness-of-fit of models conducted in the SEM framework. However, the chi-square value has been reported to be sensitive to sample size, as even a negligible discrepancy between the model implied and observed sample covariance matrices is likely to result in a significant chi-square statistic, thereby leading to model rejection (Marsh et al., 2005). Hence, it is recommended to rely instead on descriptive goodness-of-fit indices. Thus, the Comparative Fit Index (CFI), the Root Mean Square Error of Approximation (RMSEA), and the Standardized Root Square Residual (SRMR) were used for the evaluation of the goodness-of-fit. According to the most widely used cutoff guidelines fit indices of CFI ≥ 0.90, TLI ≥ 0.90, RMSEA ≤ 0.06, SRMR ≤ 0.08 are considered as acceptable to well-fitting models (Hu and Bentler, 1999; McDonald and Ho, 2002; Kline, 2005; Hair et al., 2010). However, Hu and Bentler (1999) point out that CFI and TLI values above 0.95 are recommended.

Measurement invariance was assessed by testing hierarchically organized multigroup CFA models, adding more equality constraints with every consecutive model. For that, invariance was tested by factor structure (configural), factor loadings (metric), and indicator/item intercepts (scalar) across countries. Models were compared with the descriptive goodness-of-fit indices as the chi-square difference tests tend to be overly sensitive for trivial misfit when the sample sizes are large. According to Cheung and Rensvold (2002) (see also Chen, 2007), invariance can be assumed as long as the CFI does not decrease more than 0.01 and as long as the RMSEA does not increase by more than 0.015 between less and more restrictive models.

In addition, in order to investigate the concurrent validity, Pearson’s correlations were performed. The ASQ-S scales were correlated with the total scores of the measures of life satisfaction, self-esteem, and symptoms of depression and anxiety.

## Results

### Mean Differences in ASQ-S Scores Across Countries

The means and standard deviations for each subscale of ASQ-S are reported in Table 1. Using an ANOVA, significant country differences in four of nine scales were found. *Post hoc* tests (Tukey) revealed that German adolescents scored significantly higher on scales of stress of school attendance than Swiss and Greek adolescents, whilst the difference between Swiss adolescents and Greek adolescents was also significant. This means that attending school was more demanding for both German-speaking samples than for the Greek sample. Moreover, it was evident that the Greek adolescents reported higher levels of stress in terms of romantic relationship and peer pressure compared to the Swiss and German samples. However, there were no significant differences between the German-speaking countries. Lastly, the mean difference analyses indicated that Swiss adolescents had the highest score regarding stress in terms of future uncertainty, but this difference was only significant when the Swiss sample was compared with the Greek sample. Likewise, the differences for the total score were only visible between the Greek and Swiss samples, with the former having the highest total stress score.

### Reliability

We assessed reliability by calculating Cronbach’s α and Spearman-Brown coefficients for the full sample and for each country. As the Spearman-Brown coefficient is a more appropriate measure of reliability than Cronbach’s α for two-item scales (Eisinga et al., 2013), Spearman-Brown coefficients were calculated for the subscales School Attendance, Romantic Relationships, Future Uncertainty, and Financial Pressure. For the 9-factor solution, only moderate to low internal reliability across the country groups was found. Only three of nine subscales showed Cronbach α or Spearman-Brown coefficients above 0.70. In particular, two of three Cronbach α-values for the scale Stress of Teacher Interaction were below α = 0.60, indicative of poor internal consistency. Nevertheless, the factor Stress of School Life revealed good internal consistencies in all three countries. These results suggest that the ASQ-S is also problematic in terms of internal consistency when administered to participants of different countries. However, the values of Cronbach α for the total scores were above α = 0.93. In addition, the corrected item-factor correlations were also satisfactory (see Tables 2, 3), as all values were above *r* = 0.30, except for Item 19 (Getting along with your teachers) in the 9-factor solution (value of *r* = 0.27 for German sample vs. value of *r* = 0.15 for Greek sample).

**TABLE 2:** 9-factor structure of the adolescent stress questionnaire-shortened version (ASQ-S); standardized loadings and item-total correlations.

	CH		GER		GR	
Factors and indicators	Item	Item-total	Item	Item-total	Item	Item-total
	loading	correlation	loading	correlation	loading	correlation
**F1: Stress of Home Life**						
Item 1: Arguments at home	–	–	–	–	–	–
Item 2: Disagreement between your parents	0.86	0.71	0.91	0.77	0.74	0.60
Item 3: Disagreement between you and your mother	0.80	0.68	0.83	0.74	0.79	0.63
Item 4: Disagreement between you and your father	0.63	0.56	0.67	0.63	0.70	0.58
**F2: Stress of School Performance**						
Item 5: Having to study things you do not understand	0.57	0.47	0.61	0.48	0.50	0.42
Item 6: Teachers expecting too much from you	0.68	0.46	0.63	0.47	0.59	0.46
Item 7: Keeping up with school work	0.56	0.45	0.68	0.51	0.66	0.39
**F3: Stress of School Attendance**						
Item 8: Getting up early in the morning to go to school	0.71	0.53	0.62	0.50	0.77	0.60
Item 9: Going to school	0.74	0.53	0.81	0.50	0.79	0.60
**F4: Stress of Romantic Relationships**						
Item 10: Getting along with your boy/girl-friend	0.60	0.52	0.62	0.50	0.72	0.54
Item 11: Breaking up with your boy/girl-friend	–	–	–	–	–	–
Item 12: Making the relationship with your boy/girlfriend work	0.88	0.52	0.71	0.50	0.78	0.54
**F5: Stress of Peer Pressure**						
Item 13: Pressure to fit in with peers	0.70	0.61	0.69	0.52	0.66	0.66
Item 14: Being hassled for not fitting in	0.76	0.68	0.69	0.65	0.77	0.56
Item 15: Peers hassling you about the way you look	0.74	0.64	0.63	0.49	0.74	0.70
Item 16: Being judged by your friends	0.58	0.53	0.51	0.37	0.62	0.57
**F6: Stress of Teacher Interaction**						
Item 17: Lack of respect from teachers	0.69	0.55	0.48	0.41	0.57	0.34
Item 18: Not being listened to by teachers	0.56	0.59	0.52	0.48	0.40	0.48
Item 19: Getting along with your teachers	0.74	0.44	0.62	0.27	0.76	0.15
**F7: Stress of Future Uncertainty**						
Item 20: Concern about your future	–	-	–		–	–
Item 21: Having to make decisions about future work or education	0.59	0.40	0.65	0.41	0.78	0.55
Item 22: Putting pressure on yourself to meet your future goals	0.68	0.40	0.64	0.41	0.70	0.55
**F8: Stress of School/Leisure Conflict**						
Item 23: Not getting enough time for leisure	0.66	0.51	0.50	0.38	0.56	0.45
Item 24: Not enough time for activities outside of school hours	0.62	0.48	0.69	0.50	0.50	0.35
Item 25: Having too much homework	0.73	0.51	0.60	0.40	0.70	0.48
**F9: Stress of Financial Pressure**						
Item 26: Not enough money to buy the things you need	0.78	0.55	0.76	0.67	0.81	0.64
Item 27: Not enough money to buy the things you want	0.70	0.55	0.90	0.67	0.80	0.64

*All loadings were positive.*

**TABLE 3:** 6-factor structure of the adolescent stress questionnaire-shortened version (ASQ-S); standardized loadings and item-total correlations.

	CH		GER		GR	
Factors and indicators	Item	Item-total	Item	Item-total	Item	Item-total
	loading	correlation	loading	correlation	loading	correlation
**F1: Stress of Home Life**						
Item 1: Arguments at home	–	–	–	–	–	–
Item 2: Disagreement between your parents	0.86	0.71	0.90	0.77	0.74	0.60
Item 3: Disagreement between you and your mother	0.81	0.68	0.84	0.74	0.79	0.63
Item 4: Disagreement between you and your father	0.63	0.56	0.68	0.63	0.70	0.58
**F2: Stress of School Life**						
Item 5: Having to study things you do not understand	0.56	0.41	0.60	0.54	0.52	0.45
Item 6: Teachers expecting too much from you	0.64	0.56	0.63	0.67	0.62	0.52
Item 7: Keeping up with school work	0.56	0.45	0.62	0.52	0.67	0.54
Item 8: Getting up early in the morning to go to school	0.50	0.51	0.50	0.48	0.51	0.50
Item 9: Going to school	0.50	0.46	0.59	0.59	0.53	0.58
Item 17: Lack of respect from teachers	0.59	0.58	0.40	0.37	0.48	0.36
Item 18: Not being listened to by teachers	0.64	0.54	0.57	0.54	0.71	0.61
Item 19: Getting along with your teachers	0.53	0.51	0.55	0.45	0.36	0.34
Item 23: Not getting enough time for leisure	0.63	0.53	0.50	0.39	0.50	0.46
Item 24: Not enough time for activities outside of school hours	0.59	0.44	0.66	0.60	0.49	0.44
Item 25: Having too much homework	0.71	0.62	0.59	0.58	0.63	0.59
**F3: Stress of Romantic Relationships**						
Item 10: Getting along with your boy/girl-friend	0.63	0.52	0.65	0.50	0.71	0.54
Item 11: Breaking up with your boy/girl-friend	–	–	–	–	–	–
Item 12: Making the relationship with your boy/girlfriend work	0.87	0.52	0.69	0.50	0.79	0.54
**F4: Stress of Peer Pressure**						
Item 13: Pressure to fit in with peers	0.71	0.61	0.72	0.52	0.67	0.66
Item 14: Being hassled for not fitting in	0.76	0.68	0.73	0.65	0.76	0.56
Item 15: Peers hassling you about the way you look	0.72	0.64	0.64	0.49	0.75	0.70
Item 16: Being judged by your friends	0.59	0.53	0.52	0.37	0.63	0.57
**F5: Stress of Future Uncertainty**						
Item 20: Concern about your future	–	–	–		–	–
Item 21: Having to make decisions about future work or education	0.60	0.40	0.66	0.41	0.78	0.55
Item 22: Putting pressure on yourself to meet your future goals	0.67	0.40	0.65	0.41	0.69	0.55
**F6: Stress of Financial Pressure**						
Item 26: Not enough money to buy the things you need	0.75	0.55	0.76	0.67	0.83	0.64
Item 27: Not enough money to buy the things you want	0.73	0.55	0.92	0.67	0.78	0.64

*All loadings were positive.*

### Factor Structure

First, for all CFAs, each measured variable was allowed to load only on the factor that it was designed to measure. This restrictive, *a priori* factor structure provided poorly fitting models in Switzerland and Germany in particular. A closer inspection showed that Item 11 (Breaking up with your boy/girlfriend) loaded low on the factor Stress of Romantic Relationships in both German-speaking samples. Thus, in the next step we excluded this item in order to get better-fitting models. Although the fit indices did get better, they were still unacceptable in all three countries.

Second, results of the modification indices in Switzerland indicated that Item 1 (Arguments at home) and Item 20 (Concern about your future) cross-loaded with one or more other manifest indicators. Moreover, the modification indices showed that Item 17 (Lack of respect from teachers), Item 18 (Not being listened to by teachers), and Item 19 (Getting along with your teachers) were correlated. That is why the models were rerun with the exclusion of two more items (1, 20) and additionally allowing for correlations between Item 17, 18, and 19, as they were the indicators of the same factor. These model modifications substantially improved the model fits in every country. Results for the models are showed in Table 4, indicating adequate fit to the data with overall statistically significant factor loadings (see Table 2). For the modified 9-factor solution in Switzerland and Germany, correlations between the factors tended to vary from *r* = 0.32 (Stress of Peer Pressure with Stress of School Attendance in the Swiss sample) to *r* = 0.97 (Stress of School Performance and Stress of Teacher Interaction in the German sample). In addition, correlations between the factors Stress of School Performance, Teacher Interaction, School/Leisure Conflict, and Future Uncertainty were greater than *r* = 0.80 to *r* = 0.90 in both German-speaking samples. These very high correlations between the factors indicated that adolescents did not differentiate between the school related stressors. Moreover, it should be noted, that the correlations between the above-mentioned school stress related factors were in Greece greater than 1.0 which is statistically not possible. Thus, even the model fits of the modificated 9-factor model were acceptable, the factor correlations suggested that the factors Stress of School Attendance, School Performance, Teacher Interaction, School/Leisure Conflict did not represent four distinct factors in all three samples.

**TABLE 4:** Evaluations of measurement invariance between Swiss, German, and Greek samples.

Model	Type of test	Compared with	χ^2^	χ^2^ scaled	df	RMSEA	CFI	TLI	SRMR	Δdf	Δχ^2^ scaled	ΔCFI	ΔRMSEA	ΔSRMR
9-factor Solution														
M1a	Switzerland		325.287	378.634	214	0.039	0.941	0.924	0.047					
M1b	Germany		336.719	375.812	214	0.042	0.932	0.912	0.054					
M1c	Greece		342.383	387.578	214	0.039	0.936	0.918	0.052					
6-factor Solution														
M2a	Switzerland		364.257	423.558	234	0.040	0.931	0.919	0.050					
M2b	Germany		354.476	401.160	234	0.040	0.933	0.921	0.056					
M2c	Greece		357.432	406.936	234	0.037	0.939	0.928	0.054					
M3	Configural invariance		1089.167	1246.987	702	0.039	0.932	0.920	0.053					
M4	Metric invariance	M3	1157.010	1315.289	738	0.040	0.927	0.918	0.061	36	69.778***	−0.005	+0.001	+0.008
M5	Scalar invariance	M4	1456.168	1646.780	774	0.050	0.881	0.872	0.067	36	328.226***	−0.046	+0.010	+0.006

***p < 0.001. M1a-M1c and M2a-M2c = estimated models for the 9-factors vs. 6-factors solution per country. M3-M5 = models of invariance testing. χ^2^ = Chi-square statistic; χ^2^ scaled = corrected by Satorra and Bentler (2001) scaling method. Df, degrees of freedom; RMSEA, Root Mean Square Error of Approximation; CFI, Comparative Fit Index; TLI, Tucker Lewis Index; SRMR, Standardized Root Mean Square Error of Approximation; Δ = Change in statistical values.

Given these insufficient solutions from the 9-factor solution, in the next step we fitted 6-factor-structure models by condensing these four school-stress-related factors into one single factor (hereafter referred to as Stress of School Life). The 6-factor solution also led to acceptable goodness-of-fit indices (see Table 4) and overall significant factor loadings greater than 0.40, except for Item 19 in Greece (see Table 3). However, the correlations between the factors Stress of School Life and Stress of Future Uncertainty remained high in all country samples (CH: *r* = 0.86, GER: *r* = 0.79, GR: *r* = 0.84).

### Measurement Invariance Across Countries

We tested the final 6-dimensional model for measurement invariance across countries (see Table 4). Using the cut-off values for CFI and RMSEA differences by Cheung and Rensvold (CFI < 0.01 and RMSEA < 0.01; 2002), configural and metric measurement invariance could be established. However, according to the same criteria, invariance was missed for the scalar model, a very rarely supported and often unrealistic invariance requirement (Millsap and Yun-Tein, 2004). Establishing partial scalar invariance was also not possible.

### Concurrent Validity

We tested concurrent validity by examining correlations with measures of life satisfaction, self-esteem, depression, and anxiety. There was good concurrent validity: Scores on the ASQ-S were associated with scores on existing measures of life satisfaction, self-esteem, depression and anxiety (*r*s = −0.14 to 0.45, all *p* < 0.01; see Tables 5, 6), indicating adequate concurrent validity.

**TABLE 5:** Pearson’s correlations between the adolescent stress questionnaire (ASQ-S) scores (9-factors solution) and anxiety, depression, self-esteem and life satisfaction measures.

Factor (F)										
Variable	F1	F2	F3	F4	F5	F6	F7	F8	F9	Total ASQ-S (9 Factors)
ASQ-S										
F1: Home Life	1									0.71
F2: School Performance	0.41	1								0.74
F3: School Attendance	0.26	0.48	1							0.56
F4: Romantic Relationships	0.33	0.24	0.18	1						0.55
F5: Peer Pressure	0.55	0.43	0.30	0.41	1					0.77
F6: Teacher Interaction	0.43	0.54	0.38	0.26	0.47	1				0.71
F7: Future Uncertainty	0.59	0.49	0.29	0.24	0.54	0.44	1			0.71
F8: School/Leisure Conflict	0.42	0.59	0.46	0.35	0.47	0.50	0.48	1		0.77
F9: Financial Pressure	0.36	0.40	0.35	0.31	0.44	0.39	0.35	0.47	1	0.63
Anxiety	0.34	0.31	0.28	0.19	0.32	0.37	0.37	0.34	0.26	0.44
Depression	0.44	0.33	0.27	0.24	0.40	0.40	0.44	0.38	0.33	0.51
Self-esteem	–0.30	–0.21	–0.23	–0.21	–0.29	–0.22	–0.30	–0.20	–0.20	–0.34
Life satisfaction	–0.29	–0.20	–0.19	–0.15	–0.16	–0.26	–0.27	–0.22	–0.23	–0.31

**N* = 1,071. All correlation coefficients were significant, *p* < 0.001. Factors: F1 = Home Life; F2 = School Performance; F3 = School Attendance; F4 = Romantic Relationships; F5 = Peer Pressure; F6 = Teacher Interaction; F7 = Future Uncertainty; F8 = School/Leisure Conflict; F9 = Financial Pressure.*

**TABLE 6:** Pearson’s correlations between the adolescent stress questionnaire (ASQ-S) scores (6-factors solution) and anxiety, depression, self-esteem and life satisfaction measures.

Factor (F)							
Variable	F1	F2	F3	F4	F5	F6	Total ASQ-S (6 Factors)
ASQ-S							
F1: Home Life	1						0.60
F2: School Life	0.48	1					0.72
F3: Romantic Relationships	0.33	0.33	1				0.44
F4: Peer Pressure	0.55	0.52	0.41	1			0.74
F5: Future Uncertainty	0.59	0.53	0.24	0.54	1		0.60
F6: Financial Pressure	0.36	0.51	0.31	0.44	0.35	1	0.58
Anxiety	0.34	0.39	0.19	0.32	0.37	0.26	0.27
Depression	0.44	0.42	0.24	0.40	0.45	0.33	0.32
Self-esteem	–0.31	–0.27	–0.20	–0.29	–0.30	–0.19	–0.19
Life satisfaction	–0.29	–0.28	–0.15	–0.16	–0.27	–0.23	–0.15

**N* = 1,071. All correlation coefficients were significant, *p* < 0.001. Factors: F1 = Home Life; F2 = School Life; F3 = Romantic Relationships; F4 = Peer Pressure; F5 = Future Uncertainty; F6 = Financial Pressure.*

## Discussion

The aim of the present study was to validate a German and a Greek version of the ASQ-S (Anniko et al., 2018) and to evaluate their psychometric properties. Specifically, we addressed the following objectives: (a) the factor structure was analyzed, (b) measurement invariance across countries was tested, and (c) the reliability and concurrent validity of the questionnaire was assessed. Our results revealed that the suggested 9-dimensional shortened version of German and Greek ASQ (Anniko et al., 2018) was problematic in terms of factorial structure and internal consistency but showed satisfying concurrent validity. Thus, our results led to a new 6-factor structure, which demonstrated scales with adequate to high internal consistency, acceptable structure fit in all three countries, and form invariance across those countries.

First, using three different samples from Switzerland, Germany, and Greece in different CFA analyses, we tried to replicate the excellent model fit for the 9-dimensional ASQ-S model reported by Anniko et al. (2018). However, the results of the CFAs showed that the 9-factor structure suggested by Anniko et al. (2018), could not be replicated in our three samples. Neither the German version (validated in two different German-speaking countries Switzerland and Germany) nor the Greek version showed satisfying model fits for the CFAs conducted. Given that, in the next steps our analyses led to a new factor structure, consisting of 24 items and six correlated factors/dimensions. More specifically, adequately fitting models could be established by deleting three items and permitting correlations supported by theoretical assumptions for three other items. For example, the Item 11 (Breaking up with your boy/girlfriend) is presumably not relevant for the Swiss and German sample because of cultural expectations about dating and having romantic relationships in this age-range. This is also reflected in the adolescents’ responses: It could be shown that the scale for Stress of Romantic Relationship has considerably lower mean scores compared to other subscales. Thus, it is plausible that cultural differences explain the reported results. Similar problems with the scale of Stress of Romantic Relationship were also demonstrated in another validation study by Sotardi and Watson (2019) for the 56-item version of the ASQ from New Zealand. Moreover, these authors also reported cross-loadings of items from scales of School Performance, Teacher Interaction, and School Attendance which underline the plausibility that sources of stress experienced by adolescents in different school domains are closely related to each other. This fact lends support to our approach of condensing all school-related factors to one single factor we call Stress of School Life.

The results concerning the new factor structure contradict the results of previous studies showing good structural validity for the ASQ-S (Anniko et al., 2018; McKay et al., 2019) and the ASQ (e.g., Byrne et al., 2007; Lima et al., 2017, Tanış, 2019) as well, though they align with the results of some other international validation studies (Moksnes et al., 2010; De Vriendt et al., 2011) which also point to problems in terms of factorial structure. As regards content, the fact that the suggested 9-factor structure could not be replicated with our Swiss, German, and Greek samples suggests that the factor structures are not comparable across different countries and can be seen as indicative of different underlying stress meanings and perceptions of stress experience.

Another critical point is that the factor correlations between the scales of Stress of School Performance, School Attendance, School/Leisure Conflict, Teacher Interaction, and Future Uncertainty for the 9-factor structure vs. the factor correlations between Stress of School Life and Future Uncertainty for the 6-factor structure were too high across all countries, signaling that these four to five scales did not reflect thematically well-distinguished dimensions. Even though these highly correlated dimensions make sense–since all items represent somewhat content-related and similar concerns about school issues–from our point of view they are still questionable. Most previous studies of the short forms of ASQ did not report whether this problem was also evident in their own studies, but Anniko et al. (2018) also points to high correlations–albeit ones much lower than ours.

In terms of measurement invariance for our 6-factor solution, both the pattern of latent factors and the factor loadings were found to be equivalent across country groups, showing configural and metric invariance. However, scalar invariance (and partial scalar invariance) could not be supported, indicating that adolescents in Switzerland, Germany, and Greece use different starting points (intercepts) for scaling their responses. This means that they did not use the ordinal response categories comparably, and comparisons of mean subscale scores between the countries may thus be slightly biased. Nevertheless, the lack of scalar invariance was expected, because it is seldom supported (Chen, 2007). This is also not consistent with the results of prior studies (e.g., Anniko et al., 2018; McKay et al., 2019), which showed full measurement invariance for different age and sex groups. Yet, it is important to note that supporting scalar measurement invariance across countries is probably much more complicated than obtaining measurement invariance within groups from one country.

Even though the differences in mean values should be interpreted with caution because of the aforementioned lacking scalar invariance, *post hoc* analyses revealed significant country differences in terms of stress levels for certain stress domains. Tough, only one of the nine Cohen’s *f* values attained what Ellis (2010) describes as a large effect size. For example, German and Swiss samples reported higher levels of stress regarding school attendance, whereas the Greek sample described the stress domains related to romantic relationship and peer pressure as significantly more stress-inducing. In addition, it was also apparent that Swiss adolescents scored significantly higher than Greek adolescents regarding stress of future uncertainty. It seems to be that romantic relationships gain in importance much earlier in Greece than in both German-speaking samples. Taken together, these results support the notion that some stressors may be universal across countries while others may be more country-specific (Plunkett et al., 2000; McKay et al., 2016).

Second, we assessed the internal consistency for both factor structures and the results yielded only moderate to poor internal reliability as only three of nine vs. four of six Cronbach α-values/Spearman-Brown coefficients were greater than α = 0.70. However, the internal consistency coefficients for the total scores were above α = 0.90, indicating adequate reliability for the whole scale. As in past studies, the scale of Stress of Home Life showed the highest internal consistency in the 9-dimensional model while in the 6-dimensional model, the new created scale Stress of School Life had the highest reliability coefficient. These results align with the results of all studies using short forms of the scale (e.g., Andretta et al., 2018; Anniko et al., 2018; McKay et al., 2019; Blanco et al., 2020), arguing that Cronbach α’s are directly influenced by the number of items per scale, and that higher Cronbach α’s cannot be achieved with only few items (Streiner, 2003). This claim is also supported by the fact that the more items a scale has (e.g., Home Life, Peer Pressure, School Life), the higher the internal consistencies. The high reliabilities of both scales across the countries highlight the relevance of family and school life for adolescents’ development.

Third, we analyzed the concurrent validity of the ASQ-S and our results provided evidence for concurrent validity: All subscale scores and total scores of the ASQ-S were positively correlated with the depression and anxiety scores. This corresponds with the literature, providing support for clear associations between adolescents’ stress experience and depression and anxiety symptoms (e.g., Moksnes et al., 2010, 2013). Moreover, we found negative correlations with the scores of ASQ-S and the scores of self-esteem and life satisfaction measures, suggesting that adolescents experiencing more stress in daily life show lower self-esteem and less life satisfaction. These results are consistent with prior studies using ASQ and ASQ-S which demonstrated significant associations between adolescents’ stress experience and measures of depression, anxiety, self-esteem, and life satisfaction (e.g., Byrne et al., 2007; Lima et al., 2017; Anniko et al., 2018; McKay et al., 2019).

### Strengths, Limitations and Future Directions

To the best of our knowledge, the present study is the first cross-national validation study of the ASQ-S (Anniko et al., 2018) with a relatively large country sample. In addition, this study involves not only a validation for a translated scale in one country, but also a contemporaneous validation of one (German) translation in two different (German-speaking) countries. This may be important, as other non-replications of the stability and structure of any scales are often attributed to translation problems. Even though there is clear evidence that the translation of scales can result in different factor solutions and solutions with sub-optimal loadings (Byrne, 2008)–and this might also be true for our translations–ultimately the current study was able to show that the suggested 9-factor structure of the ASQ-S did not worked in our three samples. Nevertheless, the findings and their interpretations in this study need to be considered in relation to several limitations.

First, our study was a cross-sectional study and relied on self-reports. Therefore, it is not possible to draw causal conclusions, and the results should be interpreted with caution since social desirability biases in self-report data cannot be ruled out. Second, our data was collected in school contexts, a setting closely related to problematic scales concerning stress of school attendance, school performance, teacher interaction, and school/leisure conflict. This could also have influenced the adolescents’ response behavior. Third, it should be emphasized that the short form of the ASQ presented here contains some scales with two items per factor. Having two items to identify an underlying construct has been recognized as problematic and at least three items per construct are recommended in order to obtain reliable measures (Eisinga et al., 2013). Thus, it is important to highlight that short forms are in particular recommended for research purposes where using long forms or inventories can be a drawback because of the larger batteries.

In addition, another critical aspect, which is also visible in the long forms of the ASQ (e.g., De Vriendt et al., 2011; Lima et al., 2017), are the high correlations between various subscales. Too high correlations between factors can indicate unidimensionality of the factors. We tried to solve this problem by condensing the four school-stress-related factors into one single factor, however the problem remained. Hence future studies can address this problem more specifically.

Moreover, it is also worth considering that translation biases cannot be excluded even though established translation techniques had been applied. In particular, the item “Breaking up with my boyfriend/girlfriend” was critical, given that in German the words girlfriend and boyfriend do not exist and the German counterpart “Freund/in” can be also understood simply as a friend. This might be also an explanation for the problematic loading on the factor Stress of Romantic Relationship. Lastly, another limitation is that our sample consisted of adolescents from only seventh grade. Thus, variance in terms of age groups was not given. Future studies should involve more heterogenous samples and take these limitations into consideration.

## Conclusion

In sum, the present study revealed that the utility of the ASQ-S appears to be limited by the following concerns: The suggested 9-factor structure could not be replicated for a German and a Greek translation and the reliability also raises some concerns. However, with our slightly modified 6-factor structure, adequate and satisfying psychometric properties could be established. So, we suggest substituting the original 9-factors version and recommend our 6-factors version for research settings where long scales are difficult to implement. Nevertheless, it is worth considering that shorter instruments with fewer items often lead to a reduction in the precision of the constructs the measure is intended to capture (Anniko et al., 2018). Consequently, the ASQ-S seems to be particularly suitable for research purposes or possibly as a screening tool, but the full version should be administered if more in-depth and adequate information is needed about adolescents’ stress experience.

## Data Availability Statement

The raw data supporting the conclusions of this article will be made available by the authors after the completion of the project in 2023.

## Ethics Statement

The studies involving human participants were reviewed and approved by Ethics commission of University of Zurich and Heidelberg University of Education. Written informed consent to participate in this study was provided by the participants legal guardian/next of kin.

## Author Contributions

WK: leadership and design of the study. WK, CR, and UG: design of tasks used in the study and data collection. BE and WK: data analysis and data interpretation. BE: manuscript authorship. WK: critical revision and editing. All authors contributed to the article and approved the submitted version.

## Conflict of Interest

The authors declare that the research was conducted in the absence of any commercial or financial relationships that could be construed as a potential conflict of interest.

## References

[B1] Andretta J. R. McKay M. T. Byrne D. G. (2018). Psychometric properties of the adolescent stress questionnaire-short form scores and association with self-efficacy. *J. Psychiatry Behav. Sci.* 2:1008. 10.33582/2637-8027/1008

[B2] Anniko M. K. Boersma K. van Wijk N. P. L. Byrne D. Tillfors M. (2018). Development of a shortened version of the adolescent stress questionnaire (ASQ-S): construct validity and sex invariance in a large sample of Swedish adolescents. *Scand. J. Child Adolesc. Psychiatr. Psychol.* 6 4–15. 10.21307/sjcapp-2018-001 33520747 PMC7750703

[B3] Blanco M. J. Escobar M. Lima J. F. Byrne D. Alarcon R. (2020). Psychometric properties of a short form of the adolescent stress questionnaire (ASQ-14). *Psicothema* 32 261–267. 10.7334/psicothema2019.288 32249753

[B4] Byrne B. M. (2008). Testing for multigroup equivalence of a measuring instrument: a walk through the process. *Psicothema* 20 872–882. 18940097

[B5] Byrne D. G. Davenport S. C. Mazanov J. (2007). Profiles of adolescent stress: the development of the adolescent stress questionnaire (ASQ). *J. Adolesc.* 30 393–416. 10.1016/j.adolescence.2006.04.004 16750846

[B6] Byrne D. G. Mazanov J. (2002). Sources of stress in Australian adolescents: factor structure and stability over time. *Stress Health* 18 185–192. 10.1002/smi.940

[B7] Calvete E. Orue I. Hankin B. L. (2015). Cross-lagged associations among ruminative response style, stressors, and depressive symptoms in adolescents. *J. Soc. Clin. Psychol.* 34 203–220. 10.1521/jscp.2015.34.3.203

[B8] Chen F. F. (2007). Sensitivity of goodness of fit indexes to lack of measurement invariance. *Struct. Equ. Modeling* 14 464–504. 10.1080/10705510701301834

[B9] Cheung G. W. Rensvold R. B. (2002). Evaluating goodness-of-fit indexes for testing measurement invariance. *Struct. Equ. Modeling* 2 233–255. 10.1207/S15328007SEM0902_5 33486653

[B10] Compas B. E. Jaser S. S. Dunbar J. P. Watson K. H. Bettis A. H. Gruhn M. A. (2014). Coping and emotion regulation from childhood to early adulthood: points of convergence and divergence. *Aust. J. Psychol.* 66 71–81. 10.1111/ajpy.12043 24895462 PMC4038902

[B11] Darviri C. Legaki P. E. Chatzioannidou P. Gnardellis C. Kraniotou C. Tigani X. (2014). Adolescent stress questionnaire: reliability and validity of the greek version and its description in a sample of high school (lyceum) students. *J. Adolesc.* 37 1373–1377. 10.1016/j.adolescence.2014.10.003 25448832

[B12] De Vriendt T. Clays E. Moreno L. A. Bergman P. Vicente-Rodriguez G. Nagy E. (2011). Reliability and validity of the adolescent stress questionnaire in a sample of European adolescents-the HELENA study. *BMC Public Health* 11:717. 10.1186/1471-2458-11-717 21943341 PMC3188495

[B13] Derogatis L. R. Lipman R. S. Rickels K. Uhlenhuth E. H. Covi L. (1974). The Hopkins symptom checklist (HSCL): a self report symptom inventory. *Behav. Sci.* 19 1–15. 10.1002/bs.3830190102 4808738

[B14] Diener E. D. Emmons R. A. Larsen R. J. Griffin S. (1985). The satisfaction with life scale. *J. Pers. Assess.* 49 71–75. 10.1207/s15327752jpa4901_13 16367493

[B15] Eisinga R. Te Grotenhuis M. Pelzer B. (2013). The reliability of a two-item scale: Pearson, Cronbach, or Spearman-Brown? *Int. J. Public Health* 58 637–642. 23089674 10.1007/s00038-012-0416-3

[B16] Ellis P. D. (2010). *The Essential Guide to Effect Sizes: Statistical Power, Meta-Analysis, and the Interpretation of Research Results.* New York, NY: Cambridge University Press, 10.1017/CBO9780511761676

[B17] Enders C. K. (2013). Dealing with missing data in developmental research. *Child Dev. Perspect.* 7 27–31. 10.1111/cdep.12008

[B18] Graham J. W. (2009). Missing data analysis: making it work in the real world. *Annu. Rev. Psychol.* 60 549–576. 10.1146/annurev.psych.58.110405.085530 18652544

[B19] Hair J. F. Black W. C. Babin B. J. Anderson R. E. Tatham R. L. (2010). *Multivariate Data Analysis.* Upper Saddle River, NJ: Pearson.

[B20] Hilt L. M. McLaughlin K. A. Nolen-Hoeksema S. (2010). Examination of the response styles theory in a community sample of young adolescents. *J. Abnorm. Child Psychol.* 38 545–556. 10.1007/s10802-009-9384-3 20084450 PMC2848704

[B21] Hu L. T. Bentler P. M. (1999). Cutoff criteria for fit indexes in covariance structure analysis: conventional criteria versus new alternatives. *Struct. Equ. Modeling* 6 1–55. 10.1080/10705519909540118

[B22] Inchley J. Currie D. Budisavljevic S. Torsheim T. Jåstad A. Cosma A. (2020). *Spotlight on adolescent health and well-being.* Findings from the 2017/2018 Health Behaviour in School-aged Children (HBSC) survey in Europe and Canada. International report. Key findings. Licence: CC BY-NC-SA 3.0 IGO, Vol. 1. Copenhagen: WHO Regional Office for Europe.

[B23] Jacobs Foundation (2015). *Juvenir-Studie 4.0. Zuviel Stress - zu Viel Druck!.* Zürich: Jacobs Faoundation.

[B24] Kline R. B. (2005). *Principles and Practice of Structural Equation Modeling.* New York, NY: Guilford Press.

[B25] Lima J. F. Alarcón R. Escobar M. Fernández-Baena F. J. Muñoz ÁM. Blanca M. J. (2017). Psychometric properties of the Spanish version of the adolescent stress questionnaire (ASQ-S). *Psychol. Assess.* 29 e1–e12. 10.1037/pas0000516 28782980

[B26] Marsh H. W. Hau K.-T. Grayson D. (2005). “Goodness of fit in structural equation models,” in *Multivariate Applications Book Series. Contemporary Psychometrics: A Festschrift for Roderick P. McDonald*, eds Maydeu-Olivares A. McArdle J. J. (Mahwah, NJ: Lawrence Erlbaum Associates Publishers), 275–340.

[B27] McDonald R. P. Ho M. H. R. (2002). Principles and practice in reporting structural equation analyses. *Psychol. Methods* 7 64–82. 11928891 10.1037/1082-989x.7.1.64

[B28] McKay M. Andretta J. Perry J. (2019). The shortened version of the adolescent stress questionnaire (ASQ-S; Sweden): a validation study in United Kingdom adolescents. *Scand. J. Child Adolesc. Psychiatr. Psychol.* 7 81–87. 10.21307/sjcapp-2019-011 33520770 PMC7709939

[B29] McKay M. T. Percy A. Byrne D. G. (2016). Support for the multidimensional adolescent stress questionnaire in a sample of adolescents in the United Kingdom. *Stress Health* 32 12–19. 10.1002/smi.2570 24687846

[B30] Millsap R. E. Yun-Tein J. (2004). Assessing factorial invariance in ordered-categorical measures. *Multivariate. Behav. Res.* 39 479–515. 10.1207/S15327906MBR3903_4

[B31] Moksnes U. K. Espnes G. A. (2011). Evaluation of the Norwegian version of the adolescent stress questionnaire (ASQ−N): factorial validity across samples. *Scand. J. Psychol.* 52 601–608. 10.1111/j.1467-9450.2011.00907.x 21883256

[B32] Moksnes U. K. Espnes G. A. Haugan G. (2013). Stress, sense of coherence and emotional symptoms in adolescents. *Psychol. Health.* 29 32–49. 10.1080/08870446.2013.822868 23906224

[B33] Moksnes U. K. Moljord I. E. Espnes G. A. Byrne D. G. (2010). The association between stress and emotional states in adolescents: the role of gender and self-esteem. *Pers. Individ. Dif.* 49 430–435. 10.1016/j.paid.2010.04.012

[B34] Muthén L. K. Muthén B. O. (1998–2017). *Mplus Version 8 User’s Guide.* Los Angeles, CA: Muthén & Muthén.

[B35] Phillips S. P. Reipas K. Zelek B. (2019). Stresses, strengths and resilience in adolescents: a qualitative study. *J. Prim. Prev.* 40 631–642. 10.1007/s10935-019-00570-3 31659580

[B36] Plunkett S. W. Radmacher K. A. Moll-Phanara D. (2000). Adolescent life events, stress, and coping: a comparison of communities and genders. *Prof. Sch. Couns.* 3:356.

[B37] Rosenberg M. (1965). *Society and the Adolescent Self-Image.* Princeton, NJ: Princeton University Press.

[B38] Santrock J. W. (2004). *Life-Span Development*, 9th Edn. New York, NY: McGraw-Hill.

[B39] Satorra A. Bentler P. M. (2001). A scaled difference chi-square test statistic for moment structure analysis. *Psychometrika* 66 507–514. 10.1007/BF02296192 PMC2905175 20640194

[B40] Sawyer M. G. Arney F. M. Baghurst P. A. Clark J. J. Graetz B. W. Kosky R. J. (2000). *Mental health of young people in Australia: Child and Adolescent Component of the National Survey of Mental Health and Well-being.* Canberra: Common Wealth Department of Health and Aged Care.

[B41] Seiffge-Krenke I. (2019). What causes future-related stress in immigrant and German adolescents and how do they cope with these stressors?/was verursacht zukunftsstress bei immigrierten und deutschen jugendlichen und wie gehen beide Gruppen damit um? *Prax. Kinderpsychol. Kinderpsychiatr.* 68 606–623. 10.13109/prkk.2019.68.7.606 31711400

[B42] Seiffge-Krenke I. (2000). Causal links between stressful events, coping style, and adolescent symptomatology. *J. Adolesc.* 23 675–691. 10.1006/jado.2000.0352 11161332

[B43] Skinner E. A. Zimmer-Gembeck M. J. (2016). “Age differences and changes in ways of coping across childhood and adolescence,” in *The Development of Coping*, eds Skinner E. A. Zimmer-Gembeck M. J. (Cham: Springer), 53–62. 10.1007/978-3-319-41740-0

[B44] Sotardi V. A. Watson P. W. S. J. (2019). A sample validation of the adolescent stress questionnaire (ASQ) in New Zealand. *Stress Health* 35 3–14. 10.1002/smi.2834 30221811

[B45] Streiner D. L. (2003). Starting at the beginning: an introduction to coefficient alpha and internal consistency. *J. Pers. Assess.* 80 99–103. 12584072 10.1207/S15327752JPA8001_18

[B46] Tanış N. Ö (2019). *Adolesan Stres Ölçeği’nin Geçerlik-Güvenirlilik Çalişmasi ve SağıklıYaşam Biçimi Davranışları.* Ph. D. Thesis, Marmara University, Istanbul.

[B47] Zimmer-Gembeck M. J. van Petegem S. Skinner E. A. (2016). Emotion, controllability and orientation towards stress as correlates of children’s coping with interpersonal stress. *Motiv. Emot.* 40 178–191. 10.1007/s11031-015-9520-z

